# Reassessing the Neural Correlates of Social Exclusion: A Replication Study of the Cyberball Paradigm Using Arterial Spin Labeling

**DOI:** 10.3390/brainsci14111158

**Published:** 2024-11-20

**Authors:** Karin Labek, Roberto Viviani

**Affiliations:** 1 Institute of Psychology, University of Innsbruck, 6020 Innsbruck, Austria; 2 Department of Psychiatry and Psychotherapy III, University of Ulm, 89075 Ulm, Germany

**Keywords:** social exclusion, social pain, functional imaging

## Abstract

Background/Objectives: The cyberball paradigm has been used in numerous neuroimaging studies to elicit activation in neural substrates of social exclusion, which have been interpreted in terms of activity associated with “social pain”. The objectives of the study were to assess not only the replicability but also the specificity of the areas activated by this paradigm. Methods: Functional imaging with arterial spin labeling, an approach to image longer mental states. Results: We replicated findings of previous meta-analyses of this paradigm in the inferior frontal gyrus and ventral cingular cortex. However, these areas were also active in a watch condition (in which participants were not excluded), although less so. Conclusions: These findings relativize a simple and specific interpretation of these areas as the neural substrates of social exclusion and social pain, as in previous studies. In a broader experimental context, similar activations have been reported by neuroimaging studies when semantic disambiguation and evaluation of action goals are required, an interpretation that may also apply to the effects elicited by this paradigm.

## 1. Introduction

Social pain has been described as a distressing emotional experience resulting from rejection, loss, or social exclusion. Everyone has experienced it, making its understanding not solely a scientific question but also a universal human concern. Several paradigms have been developed to study social pain using fMRI, each providing unique insights into its neural underpinnings. Examples include trust games [1], social feedback paradigms [2,3], rejection simulations [4,5], and passive exposure to loss [6].

Among these, the cyberball paradigm [7] has been frequently investigated in neuroimaging studies. It is a virtual ball-tossing game where participants experience stages of inclusion and exclusion. In an initial practicing phase, participants view two virtual players on a computer screen exchanging a ball (watch condition). In the second phase, the ball is exchanged with the participant (play condition). In the third and final phase, the participant is unexpectedly excluded from play from the players (exclusion condition). This paradigm became notorious after Eisenberger et al. [5] showed in a functional imaging study the activation of the dorsal anterior cingulate (dACC, an area associated with pain in previous studies, [8,9]). This finding led to the proposal of the “social pain” hypothesis, which suggests that social and physical pain share common neural mechanisms/activations [5,10].

Subsequent studies carried out with this paradigm have considerably expanded the areas associated with exclusion, while often failing to replicate the original finding. Cacioppo et al. [11] failed to find significant dACC effects, instead noting activations in the ventromedial prefrontal cortex/ventral anterior cingular cortex (vmPFC/vACC), anterior insula (aI), and lateral orbitofrontal cortex/inferior frontal gyru (OFC/iFG). Vijayakumar et al. [12] replicated these results, adding to them the posterior cingulate cortex (pCC), with no significant dACC activation. They also reported activation within a left prefrontal cortex (PFC) cluster that includes the ventrolateral PFC and the lateral OFC, extending to the left inferior frontal gyrus (iFG). A meta-analysis by Rotge et al. demonstrated engagement of both ventral and dorsal subdivisions of the ACC, with the subgenual and pregenual vACC particularly associated with self-reported distress during social exclusion [13].

While the precise findings of these meta-analyses varied, they question the role of dACC for social pain, instead drawing attention to other common themes. One is the recruitment of the medial portion of the default mode network (DMN), which includes the vACC and pCC. In their recent meta-analysis, Mwilambwe-Tshilobo and Spreng [14] found that social exclusion reliably engages the medial DMN, while not reliably activating the dACC. The authors warned against attributing a function specifically related to social exclusion to the areas identified with this paradigm. A second area that is consistently reported in these meta-analyses is the iFG.

These subsequent findings justify a reassessment of the cyberball paradigm in at least two respects. The first is looking at its neural underpinnings in terms of the large-scale organization of the cortex and a broader experimental context. The DMN, which is coextensive with semantic association areas [15], is the terminal point of increasingly abstract multimodal encodings of external and internal representations [16] and differs from unimodal association areas in displaying extensive long-range connectivity [17,18,19]. Given that social cognition involves the application of high-level knowledge about situations and actions, it may not be surprising that these semantic areas are frequently active in social cognition tasks as the terminal point of progressively more abstract stimulus encoding [20,21,22] and appear in general reviews of these neuroimaging studies [23]. The iFG, which has been proposed as a point of integration between these ventral areas and the dorsal network activated by cognitive effort [24], is also consistently activated when semantic disambiguation is needed [25,26,27], and by social cognition tasks where violations of social expectations impose a reassessment of the interaction [1,28,29]. These neural substrates are the focus of the present study, using a region of interest approach to improve sensitivity.

The second is looking at effects that were not considered in the original study by Eisenberger et al. [5]. In that study, the neural substrates of social exclusion were identified by comparing the exclusion condition with the condition in which participants were actively playing the game. However, it may be argued that the initial practice condition may be an even more appropriate control condition to identify exclusion. This is because in both cases, the activity of participants is the same (watching the game), in one case becoming aware of the exclusion. In contrast, the playing condition may involve the recruitment of the resources required for active play. Since increased cognitive recruitment may depress the DMN, the question arises of the extent to which both practice and exclusion conditions share a common DMN recruitment. Therefore, broadening the scope of the contrasts considered in the analysis might provide information relevant to place the findings within the large-scale cortical organization we have just mentioned.

Finally, our study differs from most other cyberball studies in the use of arterial spin labeling (ASL) perfusion MRI. ASL provides absolute quantification of cerebral blood flow (CBF), offering a direct measure of perfusion in specific brain regions. This quantitative approach allows for comparisons across different static conditions and is especially appropriate to image long-lasting emotional states, as it does not require, as classic EPI-based imaging, relatively quick alternations of experimental and control conditions. Instead, ASL allows for planning experiments as homogenous block sessions in which participants are exposed to a homogeneous condition, as in PET designs. This is particularly appropriate here since the exclusion condition is a protracted experience, which can be compared to homogenous play and watch conditions. Despite these advantages, only a few studies have utilized ASL in conjunction with the cyberball paradigm [30].

## 2. Materials and Methods

### 2.1. Recruitment and Image Acquisition

The study was conducted at the Psychiatry and Psychotherapy Clinic of the University of Ulm, Germany, after approval by the Ethical Review Board. Healthy volunteers (N = 27) were recruited from the local university. All participants were recruited through fliers distributed in the city of Ulm. Exclusion criteria were medical, neurological, or psychiatric disorders. One participant did not complete the study, giving a final sample of N = 26 participants (16 females, mean age 24.6, standard deviation 6.1).

Magnetic resonance imaging data were acquired using the ASL sequence described in ref. [31] using a 3-Tesla MAGNETOM Prisma scanner equipped with a standard 64-channel head/neck coil (Siemens, Erlangen, Germany) at the Department of Psychiatry of the University of Ulm. After positioning in the scanner, the heads of participants were padded to minimize movement artifacts during data acquisition. Participants could always communicate with the experimenter and had the option to interrupt the scanning session. Visual stimuli were presented on a 32-inch LCD screen (NordicNeuroLab AS, Bergen, Norway) positioned behind the scanner, viewed through a mirror attached to the head coil. The ASL sequence was applied with TR/TE: 4100/23.6 ms, matrix 64 × 64, field-of-view (FOV) 224 mm, pixel spacing 3.75 × 3.75 mm, slice thickness: 5 mm, 26 slices, flip angle 90°, PAT factor 2 (GRAPPA mode), bandwidth 2298 Hz/pixel, spin labeling phase 2400 ms, and post-labeling delay 1000 ms. Conversion to CBF gave a volume every 8.2 s.

### 2.2. Experimental Task

The cyberball game, programmed using the Presentation^®^ software package (Version 18.1, Neurobehavioral Systems, Inc., Berkeley, CA, USA, www.neurobs.com) was designed to simulate social inclusion and exclusion through virtual ball-tossing between three cartoon player representations. The cyberball game task consisted of three conditions that were presented in a fixed order: (1/watch condition) passive viewing, where participants watched the ball toss without interacting; (2/inclusion play condition) social inclusion, where participants actively participated by throwing the ball to one of the virtual players; and (3/exclusion condition) social exclusion, where participants initially played as in the inclusion condition but were subsequently excluded. Each scan started with an image displaying two virtual players in the upper corners of the screen and an arm symbolizing the participant located at the bottom center.

After an initial rest period of 3 s, the first throw occurred between 500 and 1000 ms after the start of the game to increase the realism of the social interaction. Each trial type, such as a left-to-right throw, consisted of eight 200 ms stimulus events, totaling 1600 ms per trial for each condition. Both the watch and inclusion conditions were limited to a duration of 2 min. The exclusion condition was extended to 2 min 30 s to ensure that only experienced exclusion could be separated from inclusion in subsequent analyses. The exclusion phase began immediately after a 20 s inclusion phase and lasted until the end of the block. Throughout the experiment, participants responded using a button box, allowing them to choose between playing as the right or left player.

### 2.3. Statistical Modeling and Analysis

Images were realigned prior to computing estimates of cerebral blood flow with Equation (1) in Wang et al. [32]. Mean realigned EPI images were used to compute estimates of registration to an MNI template, which were subsequently applied to the CBF images. Finally, the registered CBF images (resampling size: 2 mm isotropic) were smoothed with a Gaussian kernel (FWHM 8 mm).

At the first level, conditions were modeled as blocks, all comprising 14 CBF volumes, except the exclusion block, which comprised 17 CBF volumes (the first three volumes of the exclusion block, which lasted 24.6 s longer than the others, were modeled as a confounder, so as to model exclusion with a block of the same length as the other blocks and only considering when it could become clear that the participants were was being excluded). To adjust for physiological noise, the mean activity and the first 7 principal components from white matter and ventricles (8 components in total, [33]), and the mean activity from the cranial bone [33,34], were added as covariates to the model. The segments were extracted from the segmentation computed by SPM as part of the registration algorithm. To avoid partial volume effects, activity was extracted from registered volumes (i.e., voxel size 2 mm) without smoothing. Cranial bone was eroded by 1 voxel (to avoid sampling subdural space) and white matter by 2 voxels. Ventricles were selected from the CSF segment by masking it with a priori maps of ventricles from the Harvard-Oxford Cortical and Subcortical Atlas (https://fsl.fmrib.ox.ac.uk/fsl/fslwiki/Atlases, accessed on 7 August 2007). Contrasts of interest (play vs. watch, exclusion vs. play, exclusion vs. watch) were brought to the second level to account for subjects as a random factor. All effects reported in text were corrected for multiple comparisons for the whole brain using a permutation method, except for region of interest analyses. Anatomical regions of interest for the iFG and vACC were defined with the aal atlas (iFG: 1256 voxels; vACC: 1498 voxels).

Approximate power analyses may be obtained from ref. [35], considering the number of CBF estimates per subject and sample size. Estimates of power depend on the position of the region of interest, being lower at high z coordinates. The iFG and ACC, which are the regions of interest in the present study, are at intermediate positions. Depending on region, power is estimated to be at 20%, 60%, 80%, and ~100% (Figure 7 of ref. [35]). These estimates may be conservative as the present data were acquired at 3T with a recently developed sequence with favourable signal-to-noise properties [31] and made use of adjustments for physiological noise.

## 3. Results

**Figure 1:**
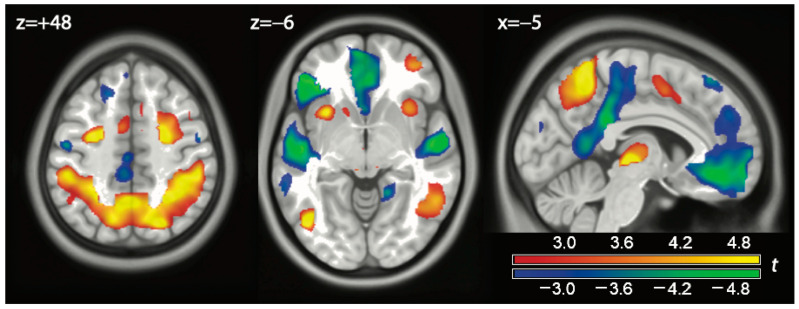
Contrast play vs. (watch or exclusion) in red-orange. In blue-green, effects in the opposite direction. Coordinates in Montreal Neurological Space. Parametric maps of *t* values displayed at *p* < 0.01, uncorrected.

We first verified that the ASL technique was implemented successfully by looking at the contrast play vs. (watch or exclude), as we expected processes recruited during active play to be identified by this contrast. As expected, dorsal cortical areas involved in attentional processing (frontal eye fields, intraparietal gyrus) were active at significant peak and cluster levels in this contrast (Figure 1, z = +48, red-yellow; 2.2–2.4 mL/(100 g min), *p* < 0.001, corrected at cluster-level; for other corrections not reported in text, see Table A1 in Appendix A). In the other direction, we observed areas that were recruited by the watch or the exclusion conditions, or both simultaneously (z = −6, blue-green, 1.8–2.0 mL/(100 g min), all significant *p* < 0.001, cluster-level corrected, Table A1). One can see that, in the medial face (x = −5 in Figure 1), they corresponded to areas of the DMN: the vACC and the pCC. This latter extended towards motor planning areas in the premotor cortex.

**Figure 2:**
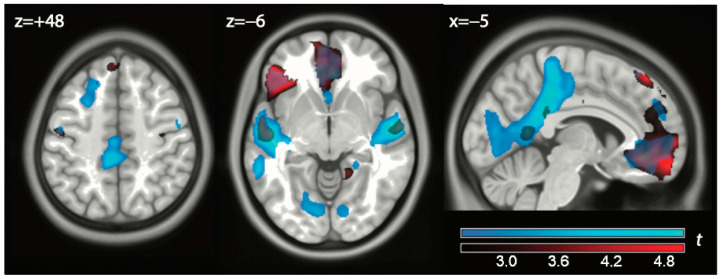
In red: contrast exclude vs. play. In light blue: contrast watch vs. play. Data displayed at *p* < 0.01, uncorrected.

We then looked at the exclusion vs. play contrast to see if we could replicate the findings of the cyberball paradigm in the literature (Figure 2, red color, and Table A2 in Appendix A). Significant effects at the peak and cluster level were detected in the iFG (2.2 mL/(100 g min), *p* = 0.002, cluster level-corrected) in a large cluster extending into the right-anterior portion of the middle temporal gyrus and in the vACC (2.1 mL/(100 g min), *p* = 0.018, cluster-level corrected; clusters #1 and #2 in Table A2). No significant effects were detected, even at uncorrected levels, in dACC. Activity in the pCC was present only at uncorrected levels and failed to reach significance.

We then looked at the contrast watch vs. play (Figure 2, light blue, and Table A3 in Appendix A). The largest effect here was in the anterior portion of the middle temporal gyrus bilaterally on the left extending posteriorly toward Heschl’s gyrus (clusters #1 and #2 in Table A3, 2.3–2.5 mL/(100 g min) all *p* < 0.001, cluster-level corrected). One can also see an intense effect in the pCC, significant at peak and cluster levels (2.2 mL/(100 g min), cluster #3, *p* = 0.001), extending dorsally into the premotor cortex. The vACC was also active at the cluster level (2.1 mL/(100 g min), cluster #4 in Table A3, *p* = 0.026, all cluster-level corrected).

One can see in Figure 2 that the effects of exclusion and watch overlapped. The main hubs of these effects (iFG, vACC, and anterior temporal lobe) were significant in both contrasts. It, therefore, appears that both contributed to the effects shown in green in Figure 1. However, there was a tendency for the exclusion contrast to involve preferentially prefrontal areas, whereas the watch contrast was most marked in posterior areas.

**Figure 3:**
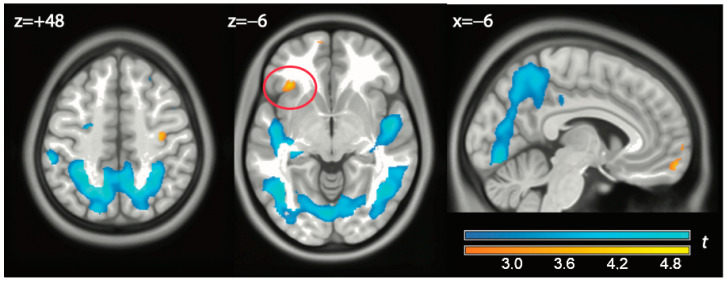
In yellow: contrast exclusion vs. watch. The red circle shows the left iFG/frontal operculum. In light blue: contrast watch vs. exclusion. Data displayed at *p* < 0.01, uncorrected.

The last contrast we looked at was the contrast exclude vs. watch (Figure 3 and Table A4 in Appendix A). This contrast tested the significance of preferential distribution in anterior and posterior areas of the effects of exclusion and watch, relative to play. This contrast recorded the higher activity in the iFG and the anterior portion of the vACC of exclusion (clusters #2 and #3 in Table A4), although this effect was significant only at the more lenient corrections for these two regions of interest (1.5 and 2.4 mL/(100 g min), *p* = 0.031 and *p* = 0.027, peak-level), in contrast to all other effects reported here. In the other direction (watch vs. exclude), we found extensive effects in visual areas extending anteriorly towards the middle temporal gyrus (clusters #4 and #9 in Table A4). These areas were located posteriorly to the common effects of exclude and watch conditions in the temporal lobe and in the pCC, but adjacent to them.

## 4. Discussion

Social pain refers to the distressing experience that results from social rejection, exclusion, or loss. It encompasses the emotional pain resulting from social disconnection, a form of pain which, as it has been argued [10], may have an evolutionary basis. However, Eisenberger’s study has faced significant criticism. Apart from the low replicability of the original dACC finding, critics have argued that activations could be related to general conflict detection or expectancy violation processes rather than social pain specifically [11]. They have also suggested that these activations might be indicative of broader negative affective processes or the processing of salient events, rather than of a specific form of pain per se. Clemens et al. [36], for example, suggested that the exclusion may differ from the watch condition because, in the former, participants are engaged in motor preparation, increasing connectivity between areas in the salience network. A similar criticism has been formulated for the evidence for the shared activations mechanism [37]. This criticism highlights the difficulty of interpreting neuroimaging results in social exclusion paradigms as the neural correlates of specific socio-emotional processes and underscore the need for careful experimental design and interpretation in this field of research.

Our findings can be brought to bear on this criticism in two respects. First, the cortical modulations observed in the exclusion condition were relatively small perfusion changes affecting a network commonly modulated by both the watch and exclusion conditions, suggesting a shared functional role. This network was partially co-extensive to the DMN but did not include areas typically found as effects of task deactivations, such as the inferior parietal junction. In the posterior part of the brain, which is deputed to the visuospatial analysis of the environment, these areas were located far from primary and secondary visual areas (i.e., the anterior part of the middle temporal gyurs/temporal poles, and pCC). In previous studies, we highlighted the role of homologous areas in the encoding of images of individuals showing negative emotion, showing that they are located at the terminal of a gradient of activity, associable to progressively abstract encodings, and consistent with a role in the application of high-level knowledge about situations and actions [21,22]. One can see that, in our data, the temporal pole and pCC activity was located anteriorly to the visual encoding activity elicited by the watch condition, consistent with high-level encoding. This functional organization is generic, in the sense that it follows general principles of encoding of sensory information [16]. It has also been shown that these high-level association areas share long-range connectivity with the DMN, in contrast to lower-level unimodal association areas [19,21]. It has, therefore, been suggested that the DMN constitutes a core cortical network with the capacity to relay information between the high-association areas of the cortex [17,18,38]. The relative specialization of the recruited areas in the watch (pCC) and in the exclusion conditions (vACC) are consistent with the prevalent role of visual information in the former, and of information about action goals [39,40,41,42] and the evaluation of aversive and appetitive environments [43,44,45,46] in the latter. However, these relative specializations are embedded in a distributed network of areas that are recruited simultaneously [47], warning against one-to-one matching of high-level functions with individual cortical areas [48].

Second, iFG function may be understood within a larger framework of numerous studies that document its modulation in social-emotional as well as cognitive paradigms. In the neuroimaging of social cognition, iFG has been noted to be active when violations of social expectations impose a reassessment of the interaction [28,29]. King-Casas et al. (2008) [1] demonstrated in a formalized strategy game that healthy controls activated the anterior insula/frontal operculum more than patients with borderline personality disorder (BPD) when attempting to restore the relationship by showing renewed cooperation efforts (see also [49,50,51]), suggesting a role in forming sophisticated representations of social interactions. This activity may be associated with the capacity of healthy individuals to form mental models of their interaction partners during the game, in contrast to BPD patients. The stimulation of this area with transcranial magnetic stimulation increases connectivity with DLPFC and appears to mitigate reports of social pain, and its activity counters the detrimental effects of negative social feedback [52]. Also in this region, however, it is possible to point out the existence of studies demonstrating a generic role in semantic disambiguation [25,26,27] that goes beyond social cognition. The anterior insular portion of this region, here more active in the exclusion than in the watch condition, has been shown to be more active when observations depart from the expected range of variation [53], explaining recruitment in social interactions when partners change their behavior, such as interrupting reciprocity in interactions [54] and social norm violations and associated negative affect [55]. In the cyberball paradigm, the exclusion condition may constitute such a violation relative to the pattern of interaction established during the play condition.

We would also like to mention the limitations of the present study. The sample size was not large. To counteract this possible limitation, we used an innovative ASL sequence with improved signal-to-noise ratio properties [31]. Given the replicatory nature of the present work, we could apply region of interest corrections for effects in the previous literature that failed to reach significance. The failed activation of dACC has been reported in the previous literature and meta-analyses. A further limitation was that no subjective responses to exclusion were collected.

In conclusion, our findings are consistent with those reported in meta-analyses of the cyberball paradigm, confirming its replicability. However, they are also consistent with those of a much broader set of studies. On the one hand, this draws attention to the relative lack of specificity of this paradigm and its findings, in line with previous criticism [11,14]. The risk is one of treating operational constructs as if they were natural entities with a specific mapping onto cortical neurobiological processes. On the other hand, it underscores the internal consistency of neuroimaging data when interpreted in an ecumenical approach, i.e., across the boundaries of traditional paradigm distinctions. As in other studies, the left iFG was recruited when violations of assumptions increased processing demands in interpreting the semantics of the social interaction.

## Data Availability

The data presented in this study are available on request from the corresponding author due to privacy restrictions, as only persons who are bound by confidentiality and authorized have permission to access the data.

## Appendix A

**Table A1:** Contrast play vs. (watch or exclusion).

Cl #	Brain Area	Coord. (mm.)	*t*	*p* (Uncorr.)	*p* (Corr.)	*k*	*p* (Cl.)
Play vs. (watch or exclusion)							
1	Left Middle Temporal Gyrus (BA 37)	−46 −68 0	8.416	<0.0001	0.000	581	0.013
	Left Culmen (BA 37)	−42 −50 −26	5.965	<0.0001	0.015		
2	Left Middle Frontal Gyrus (BA 6)	−28 −6 48	7.878	<0.0001	0.000	535	0.015
	Left Middle Frontal Gyrus (BA 6)	−26 −8 62	7.068	<0.0001	0.001		
3	Left Inferior Parietal Lobule (BA 40)	−48 −36 32	7.838	<0.0001	0.000	6504	<0.001
	Left Superior Parietal Lobule (BA 7)	−14 −68 58	6.854	<0.0001	0.003		
	Left Precuneus (BA 7)	−2 −52 54	6.841	<0.0001	0.003		
	Left Precuneus (BA 7)	−6 −54 64	6.767	<0.0001	0.004		
	Left Precuneus (BA 7)	−8 −56 54	6.479	<0.0001	0.005		
	Right Precuneus (BA 7)	22 −72 48	6.364	<0.0001	0.007		
	Right Superior Parietal (BA 7)	14 −68 60	6.065	<0.0001	0.013		
	Left Superior Parietal (BA 7)	−28 −50 68	6.058	<0.0001	0.013		
	Right Postcentral Gyrus (BA 2)	48 −34 54	5.728	<0.0001	0.024		
	Left Inferior Parietal Lobule (BA 40)	−32 −44 40	5.728	<0.0001	0.024		
	Right Inferior Parietal Lobule (BA 40)	52 −36 40	5.655	<0.0001	0.027		
	Right Superior Parietal Lobule (BA 7)	34 −50 60	5.620	<0.0001	0.029		
	Right Inferior Parietal Lobule (BA 40)	38 −46 42	5.414	<0.0001	0.043		
	Right Precuneus (BA 7)	14 −56 54	5.383	<0.0001	0.044		
	Right Inferior Parietal Lobule (BA 40)	40 −38 48	5.367	<0.0001	0.045		
	Left Precuneus (BA 7)	−26 −52 44	5.284	<0.0001	0.054		
	Right Sub-Gyral (BA 40)	40 −38 38	5.167	<0.0001	0.068		
	Right Precuneus (BA 7)	20 −62 40	5.022	<0.0001	0.091		
	Left Inferior Parietal Lobule (BA 40)	−38 −44 58	4.269	0.0001	0.332		
4	Left Posterior Thalamus	−6 −22 −2	7.201	<0.0001	0.001	250	0.050
	Right Posterior Thalamus	6 −22 −2	5.632	<0.0001	0.028		
5	Right Superior Frontal Gyrus (BA 6)	18 12 62	6.352	<0.0001	0.007	1117	0.003
	Right Temporal Pole (BA 20)	22 −4 50	6.314	<0.0001	0.008		
	Right Middle Frontal Gyrus (BA 6)	30 −6 60	6.214	<0.0001	0.010		
	Right Middle Frontal Gyrus (BA 6)	30 12 60	5.726	<0.0001	0.024		
6	Right Inf Temporal Gyrus (BA 37)	48 −52 −26	6.276	<0.0001	0.009	1046	0.004
	Right Inf Temporal Gyrus (BA 37)	60 −54 −14	5.132	<0.0001	0.072		
	Right Middle Temporal Gyrus (BA 21)	52 −50 2	5.077	<0.0001	0.081		
	Right Inf Temporal Gyrus (BA 37)	56 −58 −22	5.020	<0.0001	0.091		
	Right Fusiform Gyrus (BA 37)	38 −48 −24	4.653	<0.0001	0.178		
	Right Inf Temporal Gyrus (BA 37)	44 −46 −12	4.117	0.0002	0.412		
	Right Sup Temporal Gyrus (BA 22)	60 −46 16	4.080	0.0002	0.431		
	Right Inf Temporal Gyrus (BA 37)	48 −58 −4	4.026	0.0002	0.461		
7	Left Insula	−30 14 −4	5.496	<0.0001	0.036	176	0.077
(Watch or exclusion) vs. play							
8	Right Sup Temporal Gyrus (BA 21)	60 −10 −2	−7.350	<0.0001	0.001	1593	0.001
	Right Insula	42 −16 4	−5.886	<0.0001	0.016		
	Right Insula	38 −14 20	−4.981	<0.0001	0.098		
	Right Postcentral Gyrus (BA 43)	62 −6 18	−4.795	<0.0001	0.139		
	Right Lentiform Nucleus (Putamen)	30 −10 6	−3.768	0.0005	0.600		
9	Left Middle Frontal Gyrus (BA 47)	−48 40 −6	−7.226	<0.0001	0.001	1360	0.001
	Left Inferior Frontal Gyrus (BA 47)	−38 32 −2	−5.684	<0.0001	0.024		
	Left Middle Temporal Gyrus (BA 21)	−50 10 −30	−5.547	<0.0001	0.032		
	Left Inferior Frontal Gyrus (BA 47)	−48 28 −4	−5.145	<0.0001	0.071		
	Left Inferior Frontal Gyrus (BA 47)	−50 28 12	−4.917	<0.0001	0.111		
	Left Temporal Pole (BA 20)	−38 0 −46	−4.613	<0.0001	0.193		
	Left Temporal Pole (BA 38)	−44 24 −18	−4.118	0.0002	0.403		
10	Left Superior Temporal Gyrus (BA 22)	−48 −14 0	−6.037	<0.0001	0.011	1891	0.001
	Left Superior Temporal Gyrus (BA 22)	−60 −6 10	−5.471	<0.0001	0.037		
	Left Superior Temporal Gyrus (BA 21)	−58 −10 −2	−5.382	<0.0001	0.044		
	Left Insula	−40 −20 8	−5.280	<0.0001	0.055		
	Left Precentral Gyrus (BA 4/6)	−50 −12 44	−4.429	<0.0001	0.263		
	Left Middle Temporal Gyrus (BA20)	−50 −16 −18	−4.309	0.0001	0.313		
	Left Precentral Gyrus (BA4)	−50 −12 28	−4.280	0.0001	0.325		
	Left Superior Temporal Gyrus (BA 22)	−60 −30 4	−3.724	0.0005	0.628		
	Left Superior Temporal Gyrus (BA 22)	−62 −40 4	−3.703	0.0006	0.639		
11	Ventral Anterior Cingulum (BA 11)	0 38 −12	−5.559	<0.0001	0.032	1320	0.001
	Ventral Anterior Cingulum (BA 11)	−8 58 −22	−4.900	<0.0001	0.114		
	Left Medial Frontal Gyrus (BA 10)	−6 58 −8	−4.788	<0.0001	0.141		
	Left Superior Frontal Gyrus (BA 10)	−12 64 2	−4.272	0.0001	0.328		
	Left Anterior Cingulate (BA 25)	−2 16 −6	−3.894	0.0003	0.526		

Reported clusters with significance *p* = 0.01 or less, cluster-level corrected. Peaks reported with significance *p* = 0.001 or less, uncorrected. Peaks at minimum distance 10 mm. Cl #: cluster number; Coord. (mm.): MNI coordinates, in mm; *p* (uncorr.): significance levels, uncorrected; *p* (corr.): significance levels, peak-level corrected; *p* (cl.): significance levels, cluster-level corrected; *k*: cluster size, in 2 mm voxels.

**Table A2:** Contrast exclusion vs. play.

Cl #	Brain Area	Coord. (mm.)	*t*	*p* (Uncorr.)	*p* (Corr.)	*k*	*p* (Cl.)
Exclusion vs. play							
1	Left Inferior Frontal Gyrus (BA 47)	−50 38 −8	7.088	<0.0001	0.002	1326	0.002
					<0.001 *	*802*	<0.001 *
	Left Inferior Frontal Gyrus (BA 47)	−36 30 −4	6.091	<0.0001	0.009		
					<0.001 *		
	Left Inferior Frontal Gyrus (BA 47)	−48 28 −6	5.748	<0.0001	0.021		
					<0.001 *		
	Left Sup Temporal Gyrus (BA 38)	−48 12 −28	4.900	<0.0001	0.116		
	Left Inferior Frontal Gyrus (BA 47)	−50 28 10	4.815	<0.0001	0.133		
	Left Temporal Pole (BA 20)	−40 6 −46	4.129	0.0002	0.385		
2	Left Medial Frontal Gyrus (BA 11)	−6 60 −14	5.839	<0.0001	0.018	1031	0.004
					<0.001 *	*919*	<0.001 *
	Left Sup Frontal Gyrus (BA 10)	−12 64 2	5.405	<0.0001	0.043		
					0.001 *		
	Left Medial Frontal Gyrus (BA 11)	−2 38 −12	4.916	<0.0001	0.112		
					0.002 *		
	Left Sup Frontal Gyrus (BA 10)	−4 64 2	4.627	<0.0001	0.187		
					0.005 *		
	Right Med Frontal Gyrus (BA 11)	8 46 −8	3.873	0.0004	0.525		
Play vs. exclusion							
3	Left Mid Temporal Gyrus (BA 37)	−46 −64 4	−9.968	<0.0001	0.000	1551	0.002
	Left Fusiform Gyrus (BA 37)	−42 −50 −26	−7.078	<0.0001	0.001		
	Left Fusiform Gyrus (BA 37)	−40 −60 −22	−6.305	<0.0001	0.007		
4	Right Precuneus (BA 7)	22 −70 50	−8.361	<0.0001	0.000	10,948	<0.001
	Left Precuneus (BA 7)	−10 −58 54	−8.144	<0.0001	0.000		
	Left Postcentral Gyrus (BA 2)	−46 −34 38	−8.012	<0.0001	0.000		
	Left Sup Parietal Lobule (BA 7)	−14 −68 56	−7.688	<0.0001	0.000		
	Left Precuneus (BA 7)	−8 −54 62	−7.465	<0.0001	0.001		
	Right Precuneus (BA 7)	32 −50 50	−7.410	<0.0001	0.001		
	Right Sup Parietal Lobule (BA 7)	16 −68 58	−7.165	<0.0001	0.001		
	Left Inf Parietal Lobule (BA 40)	−30 −46 42	−7.115	<0.0001	0.001		
	Right Sup Parietal Lobule (BA 7)	34 −50 60	−7.051	<0.0001	0.001		
	Right Mid Temporal Gyrus (BA 21)	54 −52 2	−6.937	<0.0001	0.001		
	Right Inferior Occipital (BA 37)	46 −60 −14	−6.914	<0.0001	0.001		
	Right Precuneus (BA 5)	14 −56 56	−6.703	<0.0001	0.003		
	Right Precuneus (BA 5)	2 −52 56	−6.671	<0.0001	0.003		
	Right Mid Temporal Gyrus (BA 37)	46 −58 −2	−6.395	<0.0001	0.006		
	Right Precuneus (BA 7)	22 −64 38	−6.356	<0.0001	0.006		
	Left Inf Parietal Lobule (BA 40)	−36 −48 58	−6.105	<0.0001	0.009		
	Right Postcentral Gyrus (BA 2)	50 −30 54	−6.081	<0.0001	0.010		
	Right Mid Temporal Gyrus (BA 37)	56 −56 −14	−6.010	<0.0001	0.011		
	Right Inf Parietal Lobule (BA 40)	54 −34 36	−4.689	<0.0001	0.167		
	Right Mid Occipital Gyrus (BA 19)	50 −70 −14	−4.598	<0.0001	0.193		
	Right Inf Temporal Gyrus (BA 20)	60 −42 −24	−3.777	0.0005	0.584		
5	Left Middle Frontal Gyrus (BA 6)	−26 −6 48	−8.303	<0.0001	0.000	531	0.015
	Left Medial Frontal Gyrus (BA 6)	−10 −6 58	−4.351	0.0001	0.284		
6	Right Middle Frontal Gyrus (BA 6)	18 10 64	−6.126	<0.0001	0.009	998	0.004
	Right Insula	30 0 64	−5.913	<0.0001	0.014		
	Right Sup Frontal Gyrus (BA 6)	22 −4 50	−5.784	<0.0001	0.018		
	Right Sup Frontal Gyrus (BA 6)	26 6 58	−5.056	<0.0001	0.083		

* significance with correction for region of interest inferior frontal gyrus or ventral ACC. Reported clusters with significance *p* = 0.01 or less, cluster-level corrected. Peaks reported with significance *p* = 0.001 or less, uncorrected. Peaks at minimum distance 10 mm. Cl #: cluster number; Coord. (mm.): MNI coordinates, in mm; *p* (uncorr.): significance levels, uncorrected; *p* (corr.): significance levels, peak-level corrected; *p* (cl.): significance levels, cluster-level corrected; *k*: cluster size, in 2 mm voxels (in italic cluster size in region of interest).

**Table A3:** Contrast watch vs. play.

Cl #	Brain Area	Coord. (mm.)	*t*	*p* (Uncorr.)	*p* (Corr.)	*k*	*p* (Cl.)
Watch vs. play							
1	Right Sup Temporal Gyrus (BA 22)	58 −12 0	7.906	<0.0001	<0.001	1946	<0.001
2	Left Sup Temporal Gyrus (BA 21)	−42 −28 6	6.606	<0.0001	0.004	2916	<0.001
	Left Insula	−46 −16 2	6.000	<0.0001	0.013		
	Left Insula	−46 −16 18	5.810	<0.0001	0.019		
	Left Sup Temporal Gyrus (BA22)	−60 −40 4	5.474	<0.0001	0.037		
	Left Sup Temporal Gyrus (BA 22)	−60 −10 −2	5.246	<0.0001	0.056		
	Left Sup Temporal Gyrus (BA 22)	−60 −6 10	5.237	<0.0001	0.057		
	Left Sup Temporal Gyrus (BA 21)	−64 −28 8	4.680	<0.0001	0.167		
	Left Claustrum	−36 −22 −4	4.418	<0.0001	0.258		
	Left Sup Temporal Gyrus (BA 42)	−66 −22 12	4.386	<0.0001	0.270		
	Left Sup Temporal Gyrus (BA 22)	−60 0 2	4.273	0.0001	0.323		
	Left Inferior Frontal Gyrus (BA 47)	−46 32 4	3.890	0.0003	0.815		
					0.031 *	*494*	<0.001 *
	Left Mid Temporal Gyrus (BA 37)	−62 −48 −8	3.685	0.0006	0.653		
3	Left Cingulate Gyrus (BA 31)	−2 −38 40	5.640	<0.0001	0.027	1253	0.001
	Left Posterior Cingulate (BA 23)	−10 −48 22	5.175	<0.0001	0.067		
	Left Paracentral Lobule (BA 6)	−4 −34 56	5.016	<0.0001	0.091		
	Left Posterior Cingulate (BA 30)	−12 −50 20	4.829	<0.0001	0.129		
	Left Paracentral Lobule (BA 5)	−8 −36 62	4.594	<0.0001	0.193		
	Left Paracentral Lobule (BA 31)	−4 −20 48	4.002	0.0003	0.472		
	Left Posterior Cingulate (BA 17)	−10 −64 12	3.801	0.0004	0.584		
4	Left Subgenual (BA 25)	−8 40 −10	4.408	0.0001	0.464	1838	0.026
					0.008 *	*635*	<0.001 *
	Left Orbitofrontal (BA 11)	−4 28 −16	3.120	0.0023	0.999		
					0.106 *		
Play vs. watch							
5	Right Middle Frontal Gyrus (BA 6)	32 −6 60	−6.889	<0.0001	0.001	961	0.003
	Right Sup Frontal Gyrus (BA 6)	18 12 60	−6.024	<0.0001	0.011		
	Right Medial Frontal Gyrus (BA 6)	18 0 54	−5.363	<0.0001	0.041		
6	Left Middle Frontal Gyrus (BA 6)	−28 −8 48	−5.562	<0.0001	0.028	330	0.027
	Left Sub-Gyral (BA 6)	−26 −6 58	−4.749	<0.0001	0.143		
	Left Middle Frontal Gyrus (BA 6)	−36 −4 62	−4.454	<0.0001	0.243		
	Left Sup Frontal Gyrus (BA 6)	−22 −2 68	−4.072	0.0002	0.427		
7	Right Inf Parietal Lobule (BA 40)	40 −38 44	−4.755	<0.0001	0.142	850	0.004
	Right Inf Parietal Lobule (BA 40)	50 −40 40	−4.454	<0.0001	0.243		

* significance with correction for region of interest inferior frontal gyrus or ventral ACC. Reported clusters with significance *p* = 0.01 or less, cluster-level corrected. Peaks reported with significance *p* = 0.001 or less, uncorrected. Peaks at minimum distance 10 mm. Cl #: cluster number; Coord. (mm.): MNI coordinates, in mm; *p* (uncorr.): significance levels, uncorrected; *p* (corr.): significance levels, peak-level corrected; *p* (cl.): significance levels, cluster-level corrected; *k*: cluster size, in 2 mm voxels (in italic cluster size in region of interest).

**Table A4:** Contrast exclusion vs. watch.

Cl #	Brain Area	Coord. (mm.)	*t*	*p* (Uncorr.)	*p* (Corr.)	*k*	*p* (Cl.)
Exclusion vs. watch							
1	Left Superior Frontal Gyrus (BA 11)	−16 58 −18	3.936	<0.001	0.489	18	0.430
2	Left Inferior Frontal Gyrus (BA 47)	−34 28 −6	3.582	<0.001	0.699	6	0.599
					0.031 *	*50*	0.053 *
3	Left Ventromedial Prefrontal (BA 11)	−10 60 −14	3.740	<0.001	0.027 *	*67*	0.050 *
Watch vs. exclusion							
4	Left Superior Temporal Gyrus(BA 42)	−64 −34 18	−7.602	<0.001	0.001	3615	<0.001
5	Right Superior Parietal Lobule (BA5)	18 −58 62	−7.306	<0.001	0.001	2949	<0.001
6	Right Insula(BA 13)	40 −20 6	−5.307	<0.001	0.047	1220	0.003
7	Right Fusiform (BA 19)	40 −72 −20	−4.928	<0.001	0.099	1249	0.003
8	Left Superior Frontal (BA 6)	−24 −8 54	−4.466	<0.001	0.228	36	0.309
9	Left Middle Occipital Gyrus (BA 39)	−42 −76 20	−4.238	<0.001	0.331	44	0.275

* significance with correction for region of interest inferior frontal gyrus or ventral ACC. Peaks reported with significance *p* = 0.001 or less, uncorrected. Peaks at minimum distance 10 mm. Cl #: cluster number; Coord. (mm.): MNI coordinates, in mm; *p* (uncorr.): significance levels, uncorrected; *p* (corr.): significance levels, peak-level corrected; *p* (cl.): significance levels, cluster-level corrected; *k*: cluster size, in 2 mm voxels (in italic cluster size in region of interest).
